# Molecular Epidemiology of Rotavirus Strains in Symptomatic and Asymptomatic Children in Manhiça District, Southern Mozambique 2008–2019

**DOI:** 10.3390/v14010134

**Published:** 2022-01-12

**Authors:** Filomena Manjate, Eva D. João, Percina Chirinda, Marcelino Garrine, Delfino Vubil, Nélio Nobela, Karen Kotloff, James P. Nataro, Tacilta Nhampossa, Sozinho Acácio, Jacqueline E. Tate, Umesh Parashar, Jason M. Mwenda, Pedro L. Alonso, Martin Nyaga, Celso Cunha, Inácio Mandomando

**Affiliations:** 1 Centro de Investigação em Saúde de Manhiça, Maputo 1929, Mozambique; eva.joao@manhica.net (E.D.J.); percina.chirinda@manhica.net (P.C.); marcelino.garrine@manhica.net (M.G.); delfino.vubil@manhica.net (D.V.); nelio.nobela@manhica.net (N.N.); tacilta.nhampossa@manhica.net (T.N.); sozinho.acacio@manhica.net (S.A.); alonsop@who.int (P.L.A.); 2 Global Health and Tropical Medicine (GHTM), Instituto de Higiene e Medicina Tropical (IHMT), Universidade Nova de Lisboa, 1349-008 Lisbon, Portugal; ccunha@ihmt.unl.pt; 3 Center for Vaccine Development, University of Maryland School of Medicine, Baltimore, MD 21201, USA; Kkotloff@medicine.umaryland.edu; 4 Department of Pediatrics, University of Virginia School of Medicine, Charlottesville, VA 22903, USA; jpn2r@virginia.edu; 5 Instituto Nacional de Saúde, Ministério da Saúde, Marracuene 1120, Mozambique; 6 Centers for Disease Control and Prevention, Atlanta, GA 30333, USA; jqt8@cdc.gov (J.E.T.); uap2@cdc.gov (U.P.); 7 African Rotavirus Surveillance Network, Immunization, Vaccines and Development Program, World Health Organization, Regional Office for Africa, Brazzaville P.O. Box 2465, Congo; mwendaj@who.int; 8 ISGlobal, Hospital Clínic, Universitat de Barcelona, 08036 Barcelona, Spain; 9 Global Malaria Program, World Health Organization, 1211 Geneva, Switzerland; 10 Next Generation Sequencing Unit and Division of Virology, Faculty of Health Sciences, University of the Free State, Bloemfontein 9300, South Africa; NyagaMM@ufs.ac.za

**Keywords:** rotavirus A, genotypes, case-control study, Mozambique

## Abstract

Group A rotaviruses remain the leading cause of diarrhoea in children aged <5 years. Mozambique introduced rotavirus vaccine (Rotarix^®^) in September 2015. We report rotavirus genotypes circulating among symptomatic and asymptomatic children in Manhiça District, Mozambique, pre- and post-vaccine introduction. Stool was collected from enrolled children and screened for rotavirus by enzyme-immuno-sorbent assay. Positive specimens were genotyped for VP7 (G genotypes) and VP4 (P genotypes) by the conventional reverse transcriptase polymerase chain reaction. The combination G12P[8] was more frequently observed in pre-vaccine than in post-vaccine introduction, in moderate to severe diarrhoea (34%, 61/177 vs. 0, *p* < 0.0001) and controls (23%, 26/113 vs. 0, *p* = 0.0013) and mixed genotypes (36%, 24/67 vs. 7% 4/58, *p* = 0.0003) in less severe diarrhoea. We observed changes in post-vaccine compared to pre-vaccine introduction, where G3P[4] and G3P[8] were prevalent in moderate to severe diarrhoea (10%, 5/49 vs. 0, *p* = 0.0002; and 14%, 7/49 vs. 1%, 1/177, *p* < 0.0001; respectively), and in less severe diarrhoea (21%, 12/58 vs. 0, *p* = 0.003; and 24%, 14/58 vs. 0, *p* < 0.0001; respectively). Our surveillance demonstrated the circulation of similar genotypes contemporaneously among cases and controls, as well as switching from pre- to post-vaccine introduction. Continuous surveillance is needed to evaluate the dynamics of the changes in genotypes following vaccine introduction.

## 1. Introduction

Group A rotaviruses (RVA) remain the predominant viral pathogen associated with diarrhoea in children aged <5 years, particularly in developing countries [1]. Globally, in 2016, RVA accounted for an estimated 258,173,300 episodes of diarrhoea, with 128,500 associated deaths, and the majority (up to 82%, N = 104,733) occurred in sub-Saharan Africa and South Asia [2]. RVA belongs to the Reoviridae family in the genus *Rotavirus*. It is a non-enveloped virus, with the genome structure of a double-stranded RNA (dsRNA) of 11 segments, composed of six structural (VP1–VP4, VP6 and VP7) and six non-structural proteins (NSP1–NSP6) [3]. RVA is commonly classified based on the binary system of two outer capsid structural proteins, VP7 (glycoprotein G) and VP4 (protease sensitive P), which can independently stimulate the production of neutralizing antibodies throughout infection [4,5].

There are 41 G (G1 to G41) and 57 P (P[1] to P[57]) single genotypes already identified [6]. The most commonly prevalent global G and P combinations associated with diarrhoea are G1P[8], G2P[4], G3P[8], G9P[8] and G12P[8]. Additionally, rare combinations, G1P[6], G2P[6], G3P[6], G8P[4] and G9P[6], are also found [7,8,9,10,11]. In 2009, the World Health Organization (WHO) recommended the introduction of rotavirus vaccine into the immunization programs in all countries, especially those with high incidence of diarrhoea [2]. Vaccine introduction can be monitored through the implementation of surveillance systems to evaluate the trend of rotavirus burden on diarrhoeal diseases, as well as the dynamics of circulating genotypes (common or emerging) in distinct periods (pre- and post-vaccine introduction) [12,13].

Following WHO recommendations, in September 2015, Mozambique introduced the monovalent rotavirus vaccine, Rotarix^®^ (GlaxoSmithKline Biologicals, Rixensart, Belgium), into its national immunization program. Data on rotavirus burden, prior to vaccine introduction in the country, is available from the previous enteric multicentre case-control study designated Global Enteric Multicenter Study (GEMS), conducted between 2007 and 2012 in three Asian countries and four sub-Saharan African countries, including Mozambique [14]. GEMS data revealed rotavirus, *Cryptosporidium*, enterotoxigenic *Escherichia coli*-producing heat stable toxin (ST-ETEC; with or without co-expression of heat-labile enterotoxin), and Shigella as the main pathogens associated with diarrhoea among all the sites. Particularly, in Mozambique (Manhiça District), rotavirus was the main pathogen associated with diarrhoea, with an attributable fraction of 35% and 20% in children aged 0–11 months with moderate to severe (MSD) and less severe diarrhoea (LSD), respectively [14,15]. This supported the introduction of rotavirus vaccine in the national immunization program.

Therefore, to assess the impact of rotavirus vaccination on diarrhoeal hospitalizations and the prevalence of circulating genotypes, in 2015, we established the surveillance of rotavirus and other enteropathogens in children less than 5 years of age in Manhiça, within the context of diarrhoeal disease surveillance platform implementation at the Manhiça District Hospital and two other peripheral health centres across the district, among children under five years of age seeking care in these sentinel sites. Additionally, to assess the burden of the disease in the community, healthy community controls were enrolled as part of the diarrhoeal disease surveillance platform.

Sparse rotavirus genotype data are available in Mozambique, and mostly, they are from the pre-vaccine introduction period, showing that the combination G12P[8] was the most frequently detected combination in Gaza Province in 2011 [16], and G2P[4] predominated in Manhiça and Mavalane between 2012 and 2013 [17]. In addition, G3P[8] and G3P[4] were the most frequent combinations found in the post-vaccine introduction period in five sentinel sites in South, central and Northern regions of the country [18]. However, molecular data and rotavirus genotypes circulating, particularly in patients with mild symptoms and asymptomatic infections, have not yet been fully explored and remain scarce in Mozambique.

To assess the most common genotypes circulating in Manhiça District, and track possible shifting in rotavirus epidemiology and genotypes after the nationwide vaccine introduction, it is critical to monitor circulating strains and describe the impact of vaccine introduction. Thus, we aim to describe the distribution of rotavirus genotypes infecting children with diarrhoea (MSD and LSD) and their paired community controls, from pre (2008–2012) and post-vaccine (2016–2019) introduction in Manhiça District, Mozambique.

## 2. Materials and Methods

### 2.1. Site Description

Data from the pre-vaccine introduction are from the GEMS study, conducted from December 2007 to November 2012 by the Centro de Investigação em Saúde de Manhiça (CISM), at the Manhiça District Hospital (MDH) and six other peripheral health facilities (Maragra, Taninga, Ilha Josina, Nwamatibjana and Malavele), and in the households of the Manhiça community. Data from the post-vaccine introduction are from the diarrhoeal disease surveillance platform, from September 2015 to December 2019, from children recruited at MDH and two peripheral health facilities (Maragra and Xinavane) and the Manhiça community. Manhiça District is a rural area located 80 km north of Maputo Province in Southern Mozambique and has sub-tropical climate characteristics, with a predominance of two seasons, warm and rainy, observed between November and April, and cool and dry during the rest of the year [19]. The geographical and socio-demographic characteristics of the community have been described previously [19,20].

CISM has been conducting a continuous demographic surveillance system (DSS) in the district since 1996, initially involving approximately 36,000 inhabitants [19]. During the GEMS study, the DSS study area had increased, covering 80,119 inhabitants from 2007–2010 and 89,617 inhabitants during 2011–2012 [19]. The diarrhoeal surveillance platform was established within a population of 169,990 inhabitants of the study area updated in 2014, while in 2019, there was an expansion of the DSS to the entire district, covering 201,845 inhabitants [20]. Within the DSS, data from births, migrations and deaths are periodically collected. CISM is adjacent to the MDH, and since 1997, the hospital and CISM have been performing a surveillance of all paediatric visits to the outpatient department (OPD), and admissions to the wards.

### 2.2. Sample Collection

The data presented in this analysis are from stool samples previously collected in children less than five years of age with MSD, LSD and community controls without diarrhoea, which tested positive for rotavirus by ELISA at the GEMS study and the diarrhoeal disease surveillance platform. GEMS started recruiting MSD and controls from 2007–2011, with the inclusion of LSD recruitment and their matched community controls in 2012. In the diarrhoeal disease surveillance platform, from September 2015 to April 2017, only MSD children were recruited to the study and from April 2017 to December 2019, there was inclusion of LSD and community controls for both MSD and LSD cases.

The definition of diarrhoeal cases (LSD and MSD), inclusion criteria for controls enrolment, sample collection, storage and processing at GEMS was previously described [14,21,22]. The diarrhoeal disease surveillance platform used the same criteria as GEMS for the enrolment of cases and controls. Briefly, children were recruited if they were admitted with diarrhoea, defined as three or more abnormal loose stools in 24 h, requiring hospitalization and oral or intravenous rehydration for MSD cases definition. LSD cases consisted of children attended at the outpatient visits without criteria for hospitalization. The community controls for both MSD and LSD cases were randomly selected through the DSS system and were matched by age, sex and neighbourhood. Stool specimens from the health facility-based cases and community controls were collected in a container, kept on ice and sent immediately to the CISM laboratory for processing.

Diarrhoeal disease surveillance activities were interrupted from 2013 to 2014 and restarted in September 2015, coinciding with rotavirus vaccine introduction in Mozambique (September 2015). However, we considered the post-vaccine introduction period as January 2016, as the data from 2015 were sparse and it was also a transition year.

Genotypes from MSD cases and controls in the pre-vaccine introduction period are from January 2008 to November 2012, and LSD are from 2012 only. In the post-vaccine introduction period among the diarrhoeal disease surveillance platform, only MSD cases (without matched controls) were included from January 2016 to April 2017, and from April 2017 to December 2019, MSD and LSD cases and respective controls were recruited.

### 2.3. Laboratory Testing

#### 2.3.1. Rotavirus Detection and RNA Extraction

Stool samples from both studies were previously screened for rotavirus using a commercial ELISA kit (Prospect^®^ Rotavirus, Oxoid, Ltd., Hampshire, UK) as described by the manufacturer. Positive samples for rotavirus were RNA extracted by QIAamp Viral RNA mini kit protocol (Qiagen, Hilden, Germany) and were stored at −70 °C until genotyping.

#### 2.3.2. cDNA Synthesis and PCR for Genotyping G and P Genes

The same assay was used for genotyping the samples from GEMS and the diarrhoeal surveillance platform. Retrospective and prospective genotyping were performed, respectively. Briefly, 8 μL of extracted RNA was reverse-transcribed to complementary DNA (cDNA) using consensus primer pairs Con2/Con3 for VP4 (P) [23], and sBeg9/End9 for VP7 (G) [24]. The cDNA product was then used as template for a multiplex semi-nested polymerase chain reaction (PCR) in conjunction with a cocktail of specific primers for each gene segment. The VP7 encoding gene was amplified using: G1, aBT1; G2, aCT2; G3, aET3 or mG3; G4, aDT4; G8, aAT8; G9, aFT9 or mG9; G12, G12b; G10, mG10 and the common primer RVG9 as previously described [25,26,27]. In parallel, the VP4 encoding gene was amplified with P[8], 1T-1D or 1T-1v; P[4], 2T-1; P[6], 3T-1; P[9], 4T-1; P[10], 5T-1; P[11], mp11; P[14], P4943 and Con3 as common primers, as previously described [24,28]. All PCR products were analysed by electrophoresis in 2.0% agarose gels, stained with 0.5 µg/mL ethidium bromide and visualized under ultraviolet illumination in a trans-illuminator imaging gel documentation system (Bio-Rad Laboratories, Hercules, CA, USA).

### 2.4. Data Management and Statistical Analysis

Data from the GEMS study were collected on specific study forms and included clinical and epidemiological risk factors and laboratory information. This information is available in one database (STATA version 14.0, StataCorp LP, Lakeway, TX, USA). Data from the diarrhoeal surveillance platform were collected on specific study forms and included clinical and epidemiological risk factors and laboratory information. Forms were checked for completeness before being double entered in the REDCap Program (version 6.17.2, Vanderbilt University, Nashville, TN, USA). All discrepancies in data entries were solved by referring to the original forms. The data analysis was performed using the statistical packages STATA version 14.1 (StataCorp LP, College Station, TX, USA) and R studios version 3.3.3 (Integrated Development for R. RStudio, PBC, Boston, MA, USA) for graphic projection. Genotype distribution was presented as absolute (*n*) and relative frequencies (%), with the significance of differences in the proportions of the groups tested by chi-square or Fisher’s exact tests as appropriate.

Due to the large number of genotypes in distribution and graphic representation, any single or combined genotype less than 1% as well as rare single or combined genotypes equal to one observation were classified and grouped as “others”. We also created a group for any mixed G or P genotypes (more than one genotype in a single sample) as “mixed genotypes” and for partial G or P typed samples a group of “partial G/P types”. The distribution of genotypes according to the age was determined based on the three age strata established at the GEMS study: 0–11 months, 12–23 months and 24–59 months of age.

The most common genotypes observed in the MSD cases were used to assess the distribution of the clinical severity score based on the Vesikari system [29], in pre- and post-vaccine introduction periods. The scores were summarized by absolute (*n*) and relative frequencies (%) for nominal variables and median and standard deviation for continuous ones. The Kolmogorov–Smirnov test was used to observe the normality of quantitative variables. The significance of differences in proportion for nominal variables was estimated by chi-square or Fisher’s exact tests when applicable and Kruskal–Wallis was used for comparisons, as an alternative to ANOVA for continuous variables. A significance level of 5% was considered for the whole analysis.

## 3. Results

### 3.1. Overview of Stool Collection and Rotavirus Group A Characterization between 2008 and 2019

During the period of January 2008–November 2012 (pre-vaccine introduction period) and January 2016–December 2019 (post-vaccine introduction period), 5353 stool samples were collected in GEMS and in the diarrhoeal surveillance platform, among children <5 years old with diarrhoea in the health centres and among ones without diarrhoea in the Manhiça community. The overall positivity of rotavirus in the pre-vaccine introduction period accounted for 29% (393/1348) in MSD and LSD cases combined and 15% (351/2411) in controls; positivity decreased in the post-vaccine introduction period, to 25% (141/559) in cases (MSD and LSD) and 11% (113/1035) in controls (Table 1). About 89% (888/998) of the positive samples were genotyped in both periods; 11% (110/998) were not possible to genotype due to insufficient amount of stool sample for testing.

Over the total genotyped samples, 43% (381/888) were fully typed (G/P), of them 50% (192/381) in cases of MSD, 29% (109/381) in cases of LSD and 21% (80/381) in controls. At least 14% (126/888) of the samples were partially typed, with 25% (32/126) in cases of MSD, 13% (16/126) in cases of LSD and 62% (78/126) in controls; 43% (381/888) of the samples were non-typeable, 22% (84/381) of them in cases of MSD, 12% (45/381) in cases of LSD and 66% (252/381) in controls. The non-typeable samples were excluded from the analysis.

### 3.2. Distribution of Single G, P and Combined G-P Genotypes among Cases of MSD, LSD and Community Controls

The characterization of rotavirus genotypes by groups (MSD, LSD and controls) evidenced that in the G-types, G12 was significantly higher in the pre-vaccine than post-vaccine introduction period in all the three groups MSD (50%, 88/177 vs. 4%, 2/49, *p* < 0.0001), LSD (49%, 33/67 vs. 9%, 5/58, *p* < 0.0001), controls (39%, 44/113 vs. 2%, 1/46, *p* < 0.0001), while G3 and G9 were significantly more prevalent in all groups in the post-vaccine introduction period, MSD (both with *p* < 0.001), LSD (*p* < 0.0001 and *p* = 0.0054) and controls (*p* = 0.0036 and *p* = 0.0014) (Table 2).

In the P types, P[8] was significantly more frequent during the pre-vaccine than post-vaccine introduction period in MSD (57%, 101/177 vs. 33%, 16/49, *p* = 0.0042) and P[6] in LSD (34%, 23/58 vs. 10%, 6/58, *p* = 0.0031), while P[4] was significantly more frequent during the post-vaccine introduction than during the pre-vaccine introduction period in MSD (47%, 23/49 vs. 15%, 27/177, *p* < 0.0001) (Table 2).

Regarding the combinations, G12P[8] was significantly more frequent in the pre-vaccine introduction than in the post-vaccine introduction period in MSD (34%, 61/177 vs. 0, *p* < 0.0001) and controls (23%, 26/113 vs. 0, *p* = 0.0013), while mixed genotypes (35%, 24/67 vs. 7%, 4/58, *p* = 0.0003) and G12P[6] (24%, 16/67 vs. 2%, 1/58, *p* = 0.0008) were significantly prevalent in LSD in the pre-vaccine than post-vaccine introduction period, respectively. G3P[4] was significantly more prevalent in MSD and LSD in the post-vaccine than in the pre-vaccine introduction period (10%, 5/49 vs. 0, *p* = 0.0002; 20%, 12/58 vs. 0, *p* < 0.0001 respectively); similarly, G3P[8] was significantly more prevalent in MSD and LSD in the post-vaccine than in the pre-vaccine introduction period (14%, 7/49 vs. 0, *p* = 0.0002; 24%, 14/58 vs. 0, *p* < 0.0001 respectively) (Table 2).

### 3.3. Temporal Distribution of Rotavirus G/P Genotype Combinations in Cases of MSD, LSD and Community Controls from 2008 to 2019

**Figure 1:**
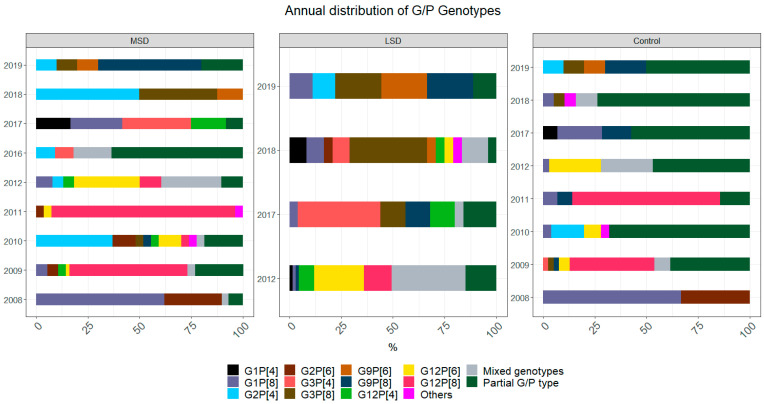
Temporal distribution of rotavirus genotype combinations found in cases and controls in children <5 years of age, in Manhiça (Maragra, Manhiça District Hospital, Taninga, Ilha Josina, Nwamatibjana, Malavele and Xinavane), Mozambique. Data represent two periods, pre-vaccine introduction, January 2008–November 2012, and post-vaccine introduction, January 2016-December 2019. No data were collected in 2013 and 2014. Colours represent each genotype combination specified, which are presented as the proportion (%) of a specific genotype combination among the total number of tested samples in each year for each group (MSD, LSD and controls). MSD—moderate to severe diarrhoea; LSD—less severe diarrhoea; controls: healthy children from the community.

The yearly distribution of common and rare genotypes combinations is shown in Figure 1. In the pre-vaccine introduction period, G1P[8] (62%, 18/29) was the most frequent combination in cases of MSD in 2008. In the following years, different combinations were recorded such as G12P[8] with 57% (32/56) in 2009 and 89% (24/27) in 2011, G2P[4] (37%, 10/27) in 2010, and G12P[6] (32%, 12/38) in 2012. Still in MSD, in the post-vaccine introduction period in 2016, most cases (64%, 7/11) were only partially genotyped. Of the remaining specimens, 2 were mixed genotypes, 1 was G2P[4], and 1 was G3P[4]. With 33% (4/12), G3P[4] was the most frequent combination in 2017 and G2P[4] (50%, 8/16) in 2018. Finally, in 2019, G9P[8] (50%, 5/10) was the most frequent genotype among all detected genotypes in MSD.

In LSD cases, mixed infections (36%, 24/67) such as G3/G12P[4] (7%, 5/67), G8/G12P[4] and G3/G8/G12P[4] [6], with 4% (3/67) each, were most frequently detected in 2012. G3P[4] (40%, 10/25) in 2017, G3P[8] (38%, 9/24) in 2018, and equal frequency of 22% (2/9) of G3P[8], G9P[8] and G9P[6] in 2019 were detected. G1P[8] (67%, 2/3) was detected in controls in 2008, while G12P[8] with 41% (16/39) and 71% (10/14) was most frequent in 2009 and 2011, respectively. G2P[4] (16%, 4/25) was more frequent in 2010, while G12P[6] (25%, 8/32) and mixed infections (25%, 8/32), such as G3/G12P[4] (13%, 4/32), were frequently detected in 2012. After the vaccine introduction period in controls, G1P[8] (21%, 3/14) was detected in 2017, mixed genotypes (11%, 2/19) in 2018 and G9P[8] (20%, 2/10) in 2019. Additionally, in 2017, 2018 and 2019, partial G/P genotypes were more frequent with 57% (8/14), 74% (14/19) and 50% (5/10), respectively.

### 3.4. Rotavirus Genotype Distribution in MSD, LSD and Community Controls According to Age Strata

In the pre-vaccine introduction period, G12P[8] was the most frequent combination in all the age strata in MSD, 0–11 months (38%, 52/137), 12–23 months (22%, 7/32) and 24–59 months (25%, 2/8). In all the age strata from LSD, mixed infections were frequent, 0–11 months (33%, 10/30), such as G3/G8/G12P[4] (20%, 7/30), 12–23 months (35%, 10/29), such as G3/G8/G12P[4]P[6] (14%, 4/29), and 24–59 months (50%, 4/8), such as G3/G12P[4] (25%, 2/8). With regard to controls, G12P[8] was frequent in children from 0–11 months (31%, 21/67) and 12–23 months (12%, 4/33), while mixed infections (23%, 3/13), such as G1/G12P[8] (8%, 1/13), were frequent in the age strata from 24 to 59 months of age.

Furthermore, in the post-vaccine introduction period, G3P[8] was frequent in the age strata from 0 to 11 months in MSD (22% 6/27) and LSD (37%, 10/27), whereas G2P[4] and G9P[8], each with 19% (3/16), were seen from 12 to 23 months in MSD. Equally, G2P[4] (33%, 2/6) was found from 24 to 59 months in MSD. Among LSD, G3P[4] was frequent in all age strata, 0–11 months (15%, 4/27), 12–23 months (32%, 6/19) and 24–59 months of age (17%, 2/12). Nonetheless, in controls, 71% (10/14) of samples were partially typed in the age strata from 0 to 11 months; contrarily, G9P[8] (17%, 4/23) was detected in children from 12 to 23 months, whereas from 24 to 59 months of age, the six samples available were partially typed (Figure S1).

### 3.5. Clinical Characteristics of Disease in the Most Common Rotavirus Genotype Combinations Found in MSD, in Pre- and Post-Vaccine Introduction Periods

Among the most commonly found G/P combined genotypes in MSD in the pre-vaccine introduction period, all children infected with G1P[8] (88%, 21/24), G2P[4] (92%,11/12), G2P[6] (100%, 15/15), G12[6] (94%, 16/17) and G12P[8] (95%, 58/61) had >3 days duration of diarrhoea and ≥ 6 episodes of diarrhoea in 24 h. Additionally, children infected with all five genotypes (G1P[8], G2P[4], G2P[6], G12P[6], G12P[8]) had a longer vomiting duration, ≥3 episodes per day (*p* < 0.05), and required intravenous rehydration, in the ascending order: 67% (16/24), 67% (10/15), 77% (47/61), 82% (14/17) and 83% (10/12), respectively (Table 3).

In the post-vaccine introduction period, we also observed that children infected with G2P[4], G3P[8], G9P[8] had diarrhoea for > 3 days and these genotypes caused >6 episodes in 24 h. Children infected with all the three genotypes (G2P[4], G3P[8], G9P[8]) suffered from vomiting with almost equal and high numbers of episodes (≥5 in 24 h) and needed intravenous dehydration: G2P[4] (100%, 10/10), G3P[8] (100%, (7/7), and G9P[8] (100%, 4/4) (Table 3).

## 4. Discussion

This is one of the few studies in the sub-Saharan Africa region that analyses changes over time of rotavirus genotypes circulating over nine years and compares pre- and post-vaccine periods. It shows a broad diversity of rotavirus genotype combinations and shift of the strains after the scale-up of rotavirus vaccine in Manhiça District, Mozambique. Strains found over the two study periods may represent those reported as common strains circulating regionally and worldwide [9]. We detected seven rotavirus G-types (G1, G2, G3, G4, G9, G10, G12), with G12 being the most frequent genotype in all groups, MSD, LSD and community controls in the pre-vaccine introduction period. Contrary to the post-vaccine introduction period, we observed a change of the most frequent G-types to G3 and G9 in MSD, LSD and controls. Our data also showed a decline of G1 in all groups, MSD, LSD and community controls from pre- to post-vaccine introduction periods, although not statistically significant. We found similar results when comparing available data from Mozambique [18], and reports from countries such as Bhutan, which had G12 in high frequency in the post-vaccine introduction period [30], and Malawi, which reported a high frequency of G3 in the post-vaccine introduction period [31].

Within VP4, P[6] was the most frequent in LSD and P[8] in MSD and controls in the pre-vaccine introduction period. This scenario remained until the post-vaccine introduction period, where P[8] was also recorded in high proportion in the three groups (MSD, LSD and controls). However, P[4] increased in proportion and was the most frequent genotype observed in LSD. The circulation of P[6] and P[8] has also been reported in high frequency elsewhere [32].

Among G/P combinations, G12P[8] was the most frequent in the pre-vaccine introduction period in MSD cases and controls. Conversely, G12P[6] was the most common combination in LSD. G12P[8] was also found to be the most frequent genotype circulating in Mozambique in 2011 in Gaza Province, while G12P[6] was most frequently detected in Maputo Province in 2012 and 2013 in the pre-vaccine introduction period [16,17]. Similar reports have shown G12P[8] as an important emerging combination worldwide, which reached a higher proportion than other common combinations [33,34]. By contrast, countries such as Ghana and Burkina Faso reported the circulation of G12P[8] after rotavirus vaccine introduction into their immunization programs [35,36].

In the post-vaccine introduction period, G3P[8], G9P[8] and G3P[4] were most frequent in LSD and MSD, while G1P[8] was prevalent in controls, although not statistically significant. These results in our MSD cases are similar to those previously found in hospital-based studies in Mozambique, which also reported G3P[8] as the most frequent combination in MSD [18]. In addition, G3P[8] was also recorded as the most frequent combination in the post-vaccine introduction in other countries such as Fiji and India [37,38]. The dynamic change in the diversity of rotavirus genotypes, we observed in the post-vaccine introduction period in our study, may not explain whether this change is related to vaccine introduction in Mozambique, because genotypes shifting may occur in countries regardless of the implementation or not of vaccine into immunization programs, demonstrating a fluctuation of genotypes within and between countries [39].

Regarding the temporal distribution of rotavirus genotypes, we observed a switching and fluctuations of genotypes year after year. In the pre-vaccination period, G1P[8] was the most frequent combination in MSD cases and controls in 2008, while G12P[8] was predominant in 2009, 2011 and 2012. In LSD in 2012, G12P[6] was found to be more prevalent. Data on the predominance of G12P[8] we have reported since 2009 are different from other studies, such as the surveillance of rotavirus in Sicily since 1985, in which they did not report G12P[8] until 2012 [40]. Nevertheless, G12P[6] was predominantly seen in 2012 after vaccine introduction into six rotavirus surveillance sites in India [41].

In the post-vaccine introduction period, G3P[4] was the most frequent combination detected in MSD and LSD in 2016 and 2017, while G3P[8] was seen in LSD in 2018 and G9P[8] in MSD, LSD and controls in 2019. Our findings are in concordance with reports of African countries such as Botswana and Kenya in which G3P[4] was found after rotavirus vaccine introduction [42,43]. However, some studies reported G3P[8] in high frequency in cases of MSD in the post-vaccine introduction period in Mozambique [18] and other African countries such as Kenya reported G3P[8] and G9P[8] [44].

No major differences were observed in the distribution of genotypes by age in both pre- and post-vaccine introduction periods. Our findings from genotypes circulating in MSD in the age strata of 12–23 months are similar to the results of a surveillance of rotavirus report in Turkey before the introduction of the vaccine [45]. However, G9P[8] was reported in children older than 24 months of age in Seoul, Republic of Korea [46].

Evaluating the clinical characteristics, we observed that children infected with G1P[8], G2P[4], G2P[6], G12P[6] and G12P[8] in the pre-vaccine and the combinations G2P[4], G3P[8] and G9P[8] in the post-vaccine introduction periods had diarrhoea for similar periods of >3 days. It is possible to hypothesize that these genotypes may cause the same disease severity. Genotypes from pre- (G1P[8], G2P[4], G2P[6], G12P[6] and G12P[8]) and post-vaccine (G2P[4], G3P[8] and G9P[8]) introduction periods are supposed to be related to severe diarrhoea symptoms as they cause more loss of stool per day (≥6). G1P[8], G2P[4], G2P[6], G3P[8], G9P[8], G12P[6] and G12P[8] are all likely to cause more frequent vomiting as well as dehydration. Previous studies associating genotypes with clinical symptoms in hospitalized children demonstrated that G12P[6] increased vomiting [47], G1P[8] was correlated with vomiting, dehydration and chronic disease [48], and G12P[8] was associated with dehydration and severity of the diarrhoea [49].

Partially typed strains, in general, constituted 62% of genotyped samples in our controls and with a significant proportion in cases of LSD (13%); contrary to our results, data of rotavirus genotypes circulating before and after vaccine introduction in Eastern and Southern African countries (2010–2015) reported low proportions of partially typed strains over time, from 6.2% in 2010 to 9.1% in 2012 and a decrease to 4.3% in 2015 [50]. The significant proportion of partially typed samples, which we found in the control group, may be explained by the fact that controls may excrete less virus than children with more severe diarrhoea, as observed by other groups [51]. In addition to this, few available studies that investigated rotavirus on LSD cases have reported a high proportion of non-typeable strains [52,53]. A study in India reported 80% and another in China 24% [52,53]. Another study on LSD and community controls reported low viral loads from both groups (LSD and community controls) [51].

We reported a high frequency of non-typeable strains, one of the limitations of this study, which may represent a failure of the PCR technique or may be, in part, due to nucleotide substitutions at the primer-binding sites, or we may have new rotavirus strains circulating in Manhiça, which are not covered by the currently used primers based on the protocol of the World Health Organization for rotavirus genotyping, which uses the primer sets from the Gouvea et al., Gentsch et al. and other authors [24,25,26,27,28], similar to what was reported among rotavirus strains from the African Rotavirus Network [54]. A previous study of whole genome sequencing of partially typed strains from Manhiça (2012–2013) suggested that the G12 and P[4] specific genotyping primers (G12b and 2T-1, respectively) should be revised to prevent incorrect typing [55]. A study from Solberg and colleagues concluded that primer mismatch may be a widespread cause of genotyping failure and might be particularly problematic in countries with greater rotavirus diversity [26,56]. Hospital-based studies in Estonia and Indonesia have also reported a high proportion of non-typeable strains, 34% and 31%, respectively [57,58]. Another study reported failure to detect mixed infections [59], while common genotypes (P[6], P[8] and G1) were undetectable [54].

The other limitation is related to the difference in the number of tested samples between the two periods. In some cases, there were small sample sizes for comparison. LSD was recruited after only one year (2012) in the pre-vaccine introduction period and in the post-vaccine introduction period, LSD and community controls were recruited after a year and half (2017) since the beginning (2015) of recruitment. The long-time break of the surveillance from pre- to post-vaccine introduction periods also limits the comparison due to unavailable samples from December 2012 to August 2015. Accordingly, we may have lost some important data regarding distribution of rotavirus genotypes early before the vaccine introduction. It also makes it difficult to attribute changes in genotype distribution to natural fluctuation, as vaccine impact on rotavirus circulating strains was difficult to assess. Despite the limitations, we were able to bring important data on the epidemiology of RVA infection in symptomatic and asymptomatic children in Manhica, and clinical presentation of MSD in each period (pre- and post-vaccine introduction periods). Although we found same genotypes circulating over the time in MSD, LSD and controls, to better investigate non-typeable strains, a new extraction protocol will be implemented to confirm the rotavirus RNA pattern (11-segmented genome) in an agarose gel electrophoresis. In addition, after rotavirus RNA pattern confirmation, we aim to apply whole genome sequencing as a genotyping strategy to characterize these strains.

## 5. Conclusions

We observed a high rotavirus genotype diversity and shift in the most genotype combinations among cases of MSD, LSD and community controls in both pre- and post-vaccine introduction periods in Manhiça District, Mozambique. Our data demonstrate circulation of similar genotypes among cases (MSD, LSD) and community controls, although it is necessary to carry out a whole genome analysis to assess whether these are the same genetic strains between symptomatic versus asymptomatic children, and the same analysis will be applied to non-typed strains for obtaining a more precise understanding of their genetic composition at the whole genome level and their evolutionary patterns. These results point out the importance of continuous surveillance of circulating rotavirus genotypes and its potential impact on the efficacy of currently administered vaccines in children, particularly in Mozambique in a rural setting such as Manhiça.

## Figures and Tables

**Table 1:** Overview of the number of collected stool samples and rotavirus group A detection among cases of MSD, LSD and controls in Manhiça District (Maragra, Manhiça District Hospital, Taninga, Ilha Josina, Nwamatibjana, Malavele and Xinavane), Mozambique, 2008–2012 and 2016–2019.

		Number of Samples Collected N			Positive Samples for Rotavirus ELISA *n* (%)			Genotyped Samples *n* (%)		
	Year	MSD	LSD	Controls	MSD	LSD	Controls	MSD	LSD	Controls
Pre-vaccine introduction	2008	343	_	344	85 (25)	_	18 (5)	56 (66)	_	9 (50)
	2009	196	_	536	72 (37)	_	90 (17)	72 (100)	_	90 (100)
	2010	143	_	416	46 (32)	_	95 (23)	41 (89)	_	92 (97)
	2011	102	_	299	43 (42)	_	54 (18)	38 (88)	_	42 (78)
	2012	133	431 ^1^	816	49 (34)	98 (23)	94 (12)	48 (98)	96 (98)	91 (97)
Subtotal	_	917	431	2411	295 (32)	98 (23)	351 (15)	255 (86)	96 (98)	324 (92)
Post-vaccine introduction	2016 ^3^	53	_	_	17 (32)	_	_	17 (100)	_	_
	2017	51	120 ^2^	372 ^2^	15 (29)	36 (30)	28 (8)	12 (80)	32 (89)	25 (89)
	2018	69	167	492	16 (23)	30 (18)	50 (10)	16 (100)	29 (97)	42 (84)
	2019	52	47	171	13 (25)	14 (29)	35 (20)	10 (77)	13 (92)	17 (49)
Subtotal	_	225	334	1035	61 (27)	80 (23)	113 (11)	55 (90)	74 (93)	84 (74)
Total	_	1142	765	3446	356 (31)	178 (23)	464 (13)	310 (87)	170 (96)	408 (87)

^1^ LSD cases recruited only in 2012 in the pre-vaccine introduction period, with no recruitment from 2008–2011. ^2^ LSD and community controls recruited from the 2017 post-vaccine introduction period. There was no active surveillance of diarrhoea cases from 2013 to 2014 and no data were included in the analysis. ^3^ Post-vaccine introduction has been considered since January 2016. MSD—moderate to severe diarrhoea; LSD—less severe diarrhoea; controls: healthy children from the community.

**Table 2:** Rotavirus single G, P and combined G-P genotypes found in cases of MSD, LSD and controls in children <5 years of age, in Manhiça (Maragra, Manhiça District Hospital, Taninga, Ilha Josina, Nwamatibjana, Malavele and Xinavane), Mozambique, 2008–2012 and 2016–2019.

	MSD			LSD			Controls		
G Genotypes	Pre-Vaccine N = 177 (*n*%)	Post-Vaccine N = 49 (*n*%)	*p*-Value	Pre-Vaccine N = 67 (*n*%)	Post-Vaccine N = 58 (*n*%)	*p*-Value	Pre-Vaccine N = 113 (*n*%)	Post-Vaccine N = 43 (*n*%)	*p*-Value
G1	26 (14)	5 (10)	0.5666	2 (3)	7 (12)	0.1069	6 (5)	6(15)	0.1404
G2	30 (17)	10 (21)	0.7264	1 (2)	2 (3)	0.8993	7 (6)	1 (2)	0.5668
G3	1 (1)	15 (31)	<0.0001	0	28 (49)	<0.0001	5 (4)	9 (21)	0.0036
G9	1 (1)	9 (18)	<0.0001	1 (2)	10 (17)	0.0054	4 (4)	9 (21)	0.0014
G12	88 (50)	2 (4)	<0.0001	33 (49)	5 (9)	<0.0001	44 (39)	1 (2)	<0.0001
Others	1 (1)	1 (2)	0.9089	1 (2)	0	1.0000	0	1 (2)	0.6145
Mixed genotypes	12 (6)	2 (4)	0.7199	21 (31)	2 (3)	0.0002	12 (11)	1 (2)	0.1768
Gx ^1^	18 (10)	5 (10)	1.0000	8 (11)	4 (7)	0.5156	35 (31)	15 (35)	0.7828
**P genotypes**									
P[4]	27 (15)	23 (47)	<0.0001	15 (22)	23 (40)	0.0577	14 (12)	7 (16)	0.7087
P[6]	37 (21)	4 (8)	0.0660	23 (34)	6 (10)	0.0031	30 (27)	6 (14)	0.1455
P[8]	101 (57)	16 (33)	0.0042	15 (22)	25 (43)	0.0224	44 (39)	15 (35)	0.7781
Others	1 (1)	1 (2)	0.9089	1 (2)	0	1.0000	6 (5)	2 (5)	1.0000
Mixed genotypes	5 (3)	0	0.5215	11 (17)	2 (3)	0.0380	5 (4)	1 (2)	0.8860
P[x] ^2^	6 (3)	5 (10)	0.1126	2 (3)	2 (4)	1.0000	14 (13)	12 (28)	0.0372
**G/P combination**									
G1P[4]	0	2 (4)	0.0661	1 (2)	2 (3)	0.8993	0	1 (2)	0.6145
G1P[8]	24 (13)	3 (6)	0.2414	1 (2)	4 (7)	0.2801	5 (5)	4 (9)	0.4335
G2P[4]	12 (6)	10 (21)	0.0100	0	1 (2)	0.9422	4 (3)	1 (2)	1.0000
G2P[6]	15 (8)	0	0.0743	0	1 (2)	0.9422	1 (1)	0	1.0000
G3P[4]	0	5 (10)	0.0002	0	12 (20)	0.0003	1 (1)	0	1.0000
G3P[8]	1 (1)	7 (14)	<0.0001	0	14 (24)	<0.0001	1 (1)	2 (5)	0.3799
G9P[6]	0	3 (6)	0.0091	0	3 (5)	0.1941	0	1 (2)	0.6145
G9P[8]	1 (1)	5 (10)	0.0013	1 (2)	5 (9)	0.1499	2 (2)	4 (9)	0.0854
G12P[4]	5 (3)	2 (4)	1.0000	5 (7)	4 (7)	0.0939	1 (1)	0	0
G12P[6]	17 (9)	0	0.0512	16 (24)	1 (2)	0.0008	12 (10)	0	0.0590
G12P[8]	61 (34)	0	<0.0001	9 (13)	0	0.0108	26 (23)	0	0.0013
Others	2 (2)	0	1.0000	0	1 (2)	0.9422	1 (1)	1 (2)	0.6145
Mixed genotypes	15 (9)	2 (4)	0.468	24 (35)	4 (7)	0.0003	11 (9)	2 (5)	0.4825
Partial G/P types	24 (14)	10 (21)	0.3366	10 (15)	6 (10)	0.6199	49 (43)	27 (64)	0.0466

^1^ Unable to determine G genotypes; ^2^ Unable to determine P genotypes; MSD—moderate to severe diarrhoea; LSD—less severe diarrhoea; controls: healthy children from the community.

**Table 3:** Clinical characteristics of disease and rotavirus genotype combinations common in MSD cases, in the pre- and post-vaccine introduction periods 2008–2012 and 2016–2019, in Manhiça (Maragra, Manhiça District Hospital, Taninga, Ilha Josina, Nwamatibjana, Malavele and Xinavane), Mozambique, 2008–2012 and 2016–2019.

Clinical Characteristics	Categories	Pre-Vaccine (January 2008–November 2012)					*p*-Value	Post-Vaccine (January 2016–December 2019)			*p*-Value
		G1P[8]	G2P[4]	G2P[6]	G12P[6]	G12P[8]		G2P[4]	G3P[8]	G9P[8]	
		N = 24 (*n*%)	N = 12 (*n*%)	N = 15 (*n* = %)	N = 17 *n*%)	N = 61 (*n*%)		N = 10 (*n*%)	N = 18 (*n*%)	N = 9 (*n*%)	
Number of days of diarrhoea	1–4 days	21 (88)	11 (92)	15 (100)	16 (94)	58 (95)	0.656	9 (90)	16 (88)	9 (100)	1.000
	5 days	2 (8)	0	0	0	1 (2)		0	1 (6)	0	
	≥6	1 (4)	1 (8)	0	1 (6)	2 (3)		1 (10)	1 (6)	0	
Maximum number of stools per day	1–3	0	0	0	0	0	NA	0	0	2 (22)	0.108
	4–5	0	0	0	0	0		5 (46)	8 (44)	1 (11)	
	≥6	24 (100)	12 (100)	15 (100)	17 (100)	61 (100)		6 (54)	10 (56)	6 (67)	
Duration of vomiting (days)	1	0	0	0	0	0	NA	3 (43)	6 (54)	0	0.166
	2	0	0	0	0	0		4 (57)	5 (46)	3 (75)	
	≥3	18 (75)	9 (75)	13 (93)	17 (100)	61 (100)	0.044	0	0	1 (25)	
Maximum number of episodes of vomiting per day	1	0	0	0	0	0	NA	1 (14)	2 (20)	0	1.000
	2–4	0	0	0	0	0		3 (43)	4 (40)	2 (50)	
	≥5	0	0	0	0	0		3 (43)	4 (40)	2 (50)	
Maximum body temperature (°C)	mean (SD)	36.1 (±0.680)	36.2 (±0.268)	36.1 (±0.425)	36.0 (±0.365)	36.2 (±0.379)	0.121 ^#^	36.7 (NA)	36.7 (±15.13)	36.6 (±0.089)	0.710
Dehydration (%)		0	0	0	0	0	NA	1 (100)	7 (54)	5 (100)	0.517
Oral rehydration *n* (%)		0	0	0	0	0	NA	1 (100)	1 (33)	0	1.000
IV rehydration *n* (%)	yes	16 (68)	10 (83)	10 (67)	14 (82)	47 (77)	0.343	10 (100)	7 (100)	4 (100)	NA

^#^ *p*-value obtained using the Kruskal–Wallis test. Abbreviations: SD—standard deviation; NA—not applicable.

## Data Availability

All relevant data are within the main text of the paper and in the Supplementary Materials.

## Supplementary Materials

The following supporting information can be downloaded at: https://www.mdpi.com/article/10.3390/v14010134/s1: Figure S1: Rotavirus genotypes distribution in MSD, LSD and community controls according to age strata.
