# Supplementary material for: Molecular Epidemiology of Rotavirus Strains in Symptomatic and Asymptomatic Children in Manhiça District, Southern Mozambique 2008–2019
Source: Viruses. 2022 Jan 12;14(1):134. doi: 10.3390/v14010134 (PMC8781303; doi:10.3390/v14010134)
Supplement: Supplementary file 1 [file viruses-14-00134-s001.zip › viruses-1513701-supplementary.pdf]

Supplementary data. Rotavirus genotypes distribution in MSD, LSD and community controls according to age strata

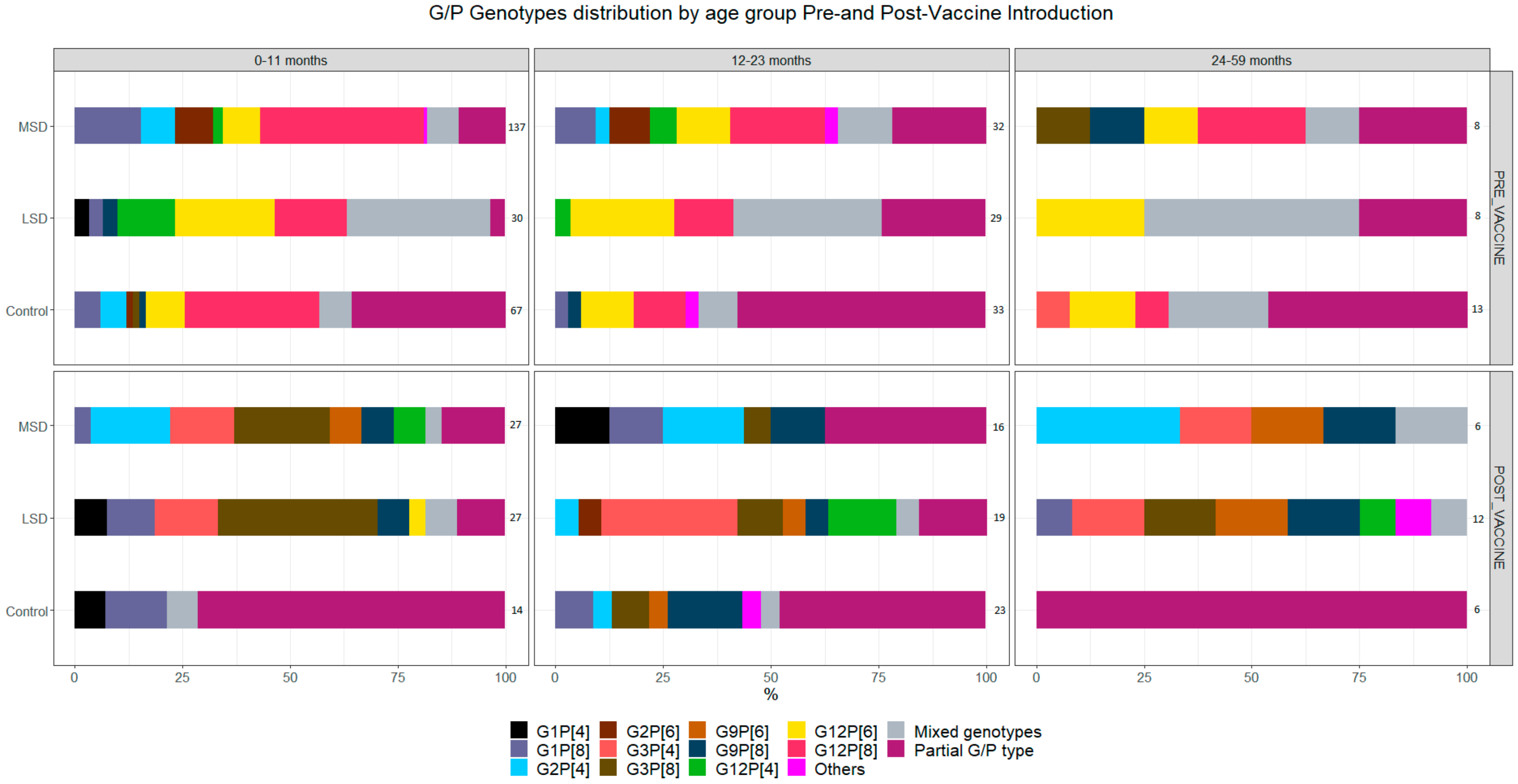

Figure S1. Distribution of rotavirus genotype combinations found in cases and controls in children < 5 years of age, among different age strata in Manhiça (Maragra, Manhiça District Hospital, Taninga, Ilha Josina, Nwamatibjana, Malavele and Xinavane), Mozambique, 2008-2012 and 2016-2019. Colours represent each genotype combination specified. Data are presented as the proportion (%) of a specific genotype among the total of tested samples in each group (MSD, LSD, and controls) and each age strata. MSD-moderate-to-severe diarrhoea; LSD-less severe diarrhoea; controls: Health children from community.
